# Efficacy of Eutectic Mixture of Local Anesthetics (EMLA) Versus Ice as Topical Anesthetics Prior to Long Buccal Nerve Blocks: A Prospective Comparative Study

**DOI:** 10.7759/cureus.45992

**Published:** 2023-09-26

**Authors:** Ashwin Pattabhi, Arun M, Saravanan Lakshmanan, Murugesan Krishnan, Santhosh P Kumar

**Affiliations:** 1 Oral and Maxillofacial Surgery, Saveetha Dental College and Hospitals, Saveetha Institute of Medical and Technical Sciences, Saveetha University, Chennai, IND

**Keywords:** ice, long buccal nerve block, topical anesthesia, dental extraction, eutectic mixture of local anesthesia

## Abstract

Background

Dental extraction is one of the common procedures in the field of dentistry. However, fear and anxiety about local anesthetic injections are unavoidable for most patients. Topical anesthetics, distraction techniques, acupuncture, and the application of heat or cold have been used in practice to alleviate injection-related pain. The specific aim of this study was to compare the pain-relieving efficacy of eutectic mixture of local anesthetics (EMLA) vs. ice during the administration of a long buccal nerve block (LBNB) before dental extraction.

Materials and Methods

In total, 20 healthy adult patients who required bilateral mandibular posterior teeth extraction under local anesthesia were enrolled. The study was conducted in the Department of Oral and Maxillofacial Surgery, Saveetha Dental College and Hospitals, Chennai, from January 2023 to May 2023. It was a prospective split-mouth study in which each participant was divided into two groups according to the operative site: Group 1, which received a 5% EMLA cream, and Group 2, which received an application of ice before administration of LBNB. There was a one-week interval between the two interventions. The levels of pain and satisfaction were assessed using the visual analogue scale (VAS) and pain perception was evaluated by the sound, eye, motor (SEM) scale. Mann-Whitney U test was applied for the statistical analysis.

Results

The study included a total of 20 participants, with 14 being male and six being female. The mean age of the study population was 42 ± 15 years. When analyzing the pain scores using the VAS, it was found that Group 1 had a mean score of 2.4 ± 0.44, while Group 2 had a mean score of 3.0 ± 0.44. This difference between the two groups was statistically significant (P = 0.001). It was also found that the mean patient satisfaction score for Group 1 was 9.8 ± 0.22 and for Group 2 was 9.2 ± 0.40 which was statistically significant (p = 0.003). Similarly, Group 1 had a mean SEM score of 1.1 ± 0.1, while Group 2 had a mean SEM score of 1.30 ± 0.46 which was also statistically significant (P =0.016).

Conclusion

The study results revealed that EMLA has a significant advantage over ice in terms of lower levels of pain, more patient satisfaction, and higher comfort levels. EMLA can be considered the first choice of topical anesthetics, however, ice is recommended in resource-constrained dental set-ups as it is cost-effective.

## Introduction

The administration of local anesthetics through injections is a distressing and anxiety-inducing aspect of dental procedures, impeding the delivery of appropriate dental care. Long buccal nerve block (LBNB), in particular, causes significant pain due to the contact of the needle with the underlying periosteum. Various criteria such as the patient's anxiety, fear perception, and individual pain tolerance during injections, lead to the exploration of topical anesthetic agents as potential solutions [1]. Various approaches have been employed to alleviate pain during local anesthetic injections, including both pharmacological and non-pharmacological methods. Non-pharmacological techniques encompass the application of topical anesthetics, distraction techniques, acupuncture, and the use of heat and cold to mitigate injection-related pain [2,3].

The most widely recommended approach for minimizing pain during local anesthesia is to apply a topical anesthetic before the injection. Research demonstrated that patients felt more comfortable when a topical anesthetic was applied beforehand, resulting in reduced anxiety and diminished pain from the needle prick [1]. The site of injection also plays a role in the perceived pain intensity. Compared to other topical agents, eutectic mixture of local anesthetics (EMLA) was found to have a better result in decreasing patient discomfort [1]. Duncan et al. have similar results in their study with a cooled refrigerant spray [2]. Ice is routinely used in rehabilitation and clinical usage because of its efficiency, economic viability, and hassle-free nature. It is believed that ice contributes to pain control by causing local anesthesia in the treated area. Literature evidence has proven that ice has the capability to lessen edema, nerve conduction velocity, cellular metabolism, and local blood flow [2].

Even though ELMA has excellent topical anesthetic properties in the skin, it has conflicting results in the oral mucosa [4]. To our knowledge, there is no study available in the literature comparing EMLA with ice before long buccal nerve blocks. Hence this comparative prospective study is designed to find out the pain-relieving efficacy of EMLA with ice prior to long buccal nerve block. The working hypothesis in the present study was that there is a difference in the pain-relieving efficacy between EMLA and ice as topical anesthetics prior to LBNB. The purpose of this study was to evaluate the levels of pain using the visual analogue scale (VAS), measure patient satisfaction through VAS ratings, and assess pain perception using the SEM (Sound, Eye, Motor) scale. Both scales were easy to administer, relatively non-cumbersome, and patient compliance was good, hence they were used in the present study. 

## Materials and methods

Study design

In total, 20 healthy adult patients who required bilateral mandibular posterior teeth extraction under local anesthesia were enrolled. The study was conducted in the Department of Oral and Maxillofacial Surgery, Saveetha Dental College and Hospitals, Chennai, from January 2023 to May 2023. The study commenced after obtaining ethical clearance from the Institutional Human Ethical Committee (IHEC/SDC/OMFS-2201/23/167). Inclusion criteria for this study were that participants had to be at least 18 years old, classified as American Society of Anesthesiologists (ASA) class I, have not taken analgesics or painkillers within the past 24 hours, have no contraindications for undergoing the extraction of posterior mandibular teeth under local anesthesia; they also had to be available after one week for a second intervention. Patients with systemic disorders, lactation, pregnancy, a history of allergies, or any concomitant pathologies were excluded from this study. Participants who met the inclusion criteria were enrolled after giving their informed consent.

Randomization and allocation process 

Participants who volunteered for this study were randomly divided into two groups, each consisting of 20 individuals, resulting in a total of 40 sites. The random number generator from the website http://www.randomization.com was used for the random assignment of the site for the group allocation. The treatment allocations for the clinician were provided in sealed, opaque envelopes, generated randomly. Once a patient agreed to participate in the trial, an envelope was opened, revealing the assigned treatment regimen. The treatment was then performed on the patient after obtaining informed written consent. One side of the treatment area of 20 sites receiving EMLA 5% cream (containing 2.5% lidocaine w/w (25 mg/g) and 2.5% prilocaine w/w (25 mg/g)) was designated as Group 1 while the contralateral side was designated as Group 2, with 20 sites subjected to the precooled ice application. The data collectors and analysts involved in the study were blinded to ensure impartiality when recording and evaluating the treatment outcomes.

Surgical technique

**Figure 1:**
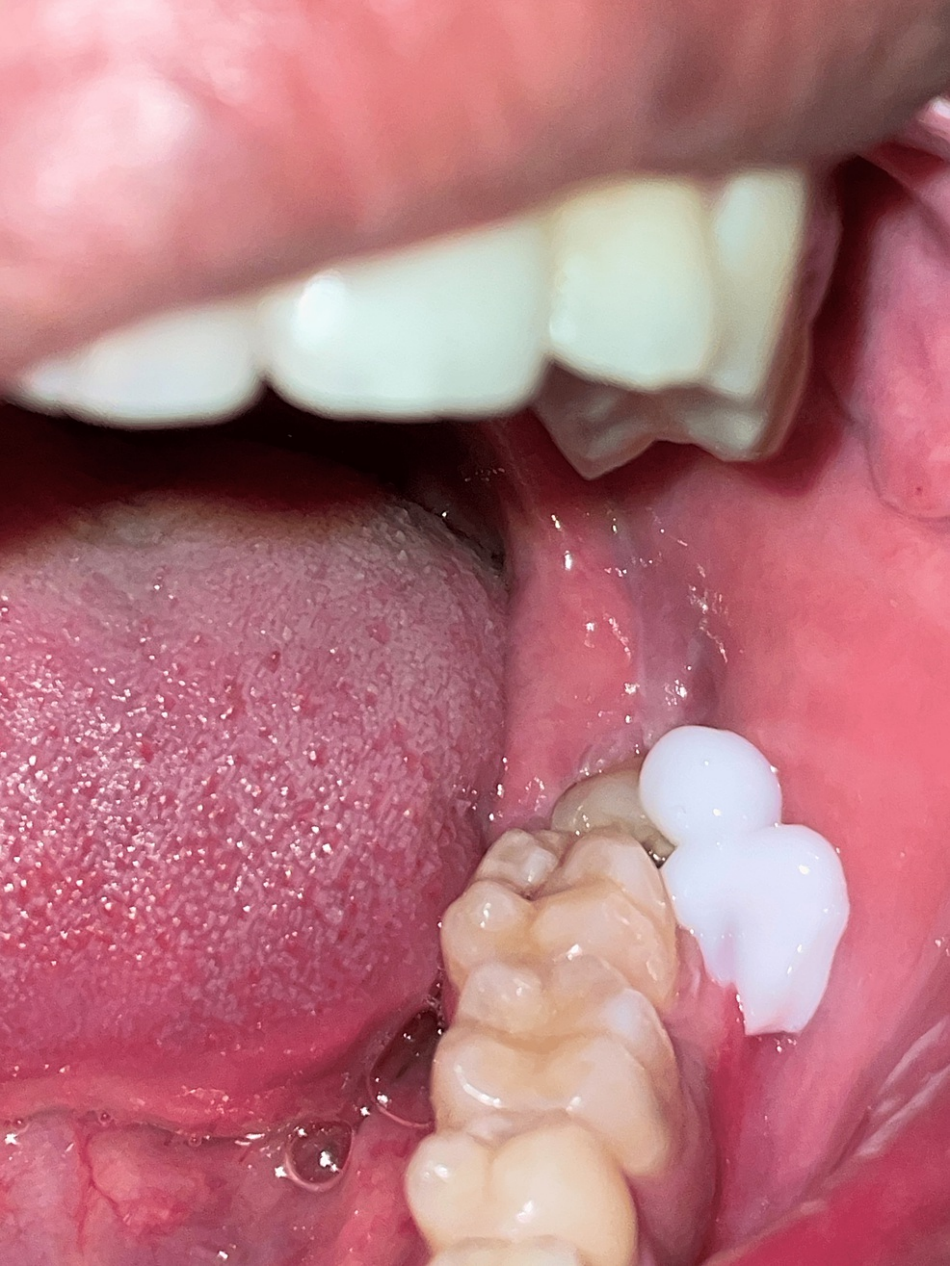
Application of EMLA prior to long buccal nerve block

**Figure 2:**
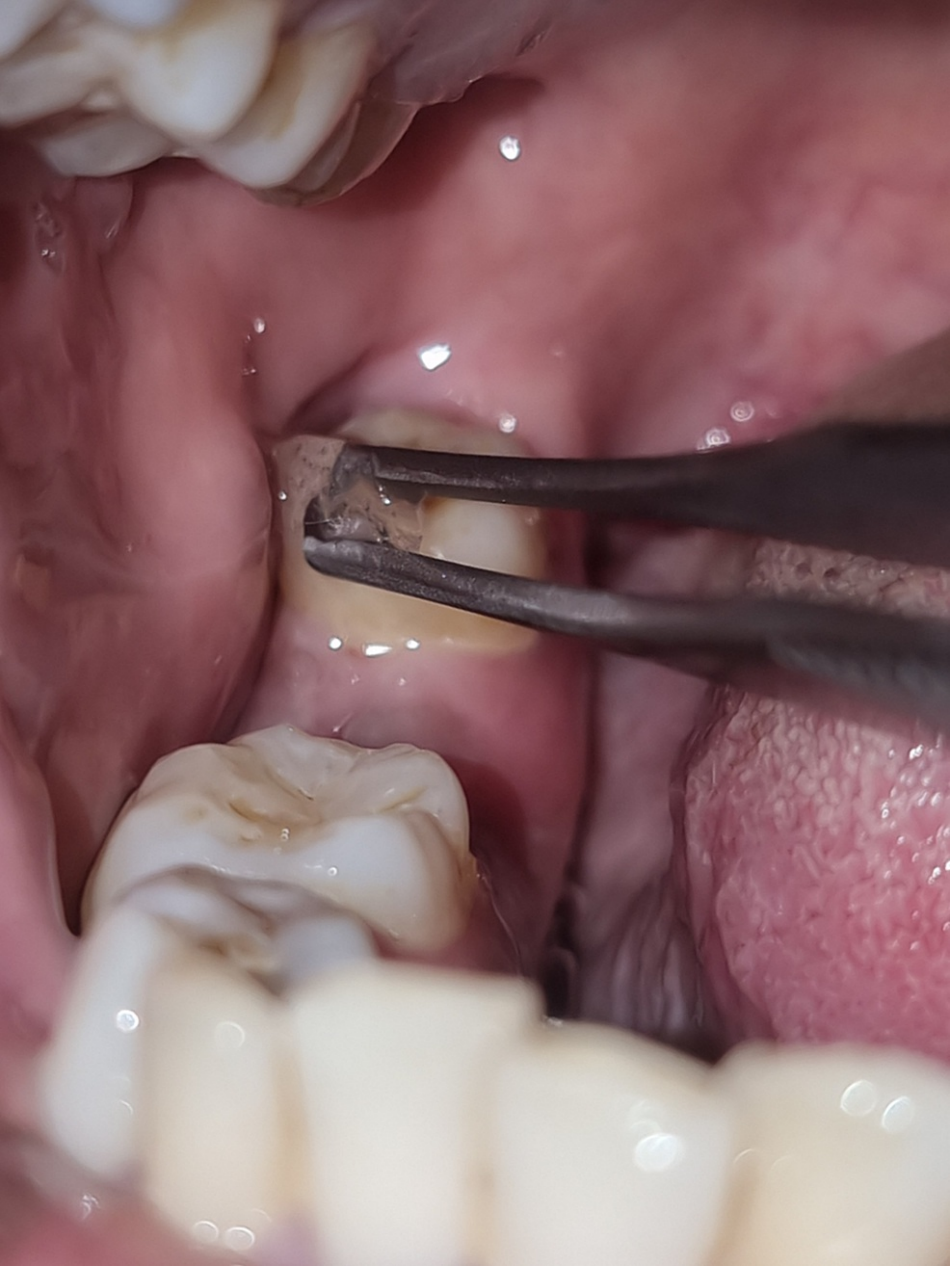
Application of Ice prior to long buccal nerve block

The patient was kept in an upright position in the dental chair to prevent the topical agent from flowing toward the soft palate and pharynx. To ensure a dry surface, the clinician used a sterilized 2x2 cm gauze to remove the saliva from the buccal mucosa distal to the second molar. Out of the total 40 sites, half were subjected to treatment with EMLA 5% cream (Figure 1), while the other half were treated with ice (Figure 2) for a duration of two minutes before administering the LBNB. After drying the mucosa 0.5mg EMLA cream was applied using a cotton applicator without any pressure. Preparation of the ice was made with water-filled straws measuring 3 cm and placed in the freezer having a temperature of -18℃. After administering precooled ice or the eutectic mixture for two minutes, local anesthetic injections were administered at all sites using a one-inch, 25-gauge needle containing 2% lignocaine and 1:80,000 adrenaline. The depth of needle penetration was 2-3 mm, and 0.5ml anesthetic solution was delivered over a period of 15 secs. The primary outcomes were recorded, and extraction of the teeth was performed under standard protocol. There was a one-week interval between the two interventions. The same protocol was used in the second setting on the contralateral side. 

Primary outcomes

**Figure 3:**
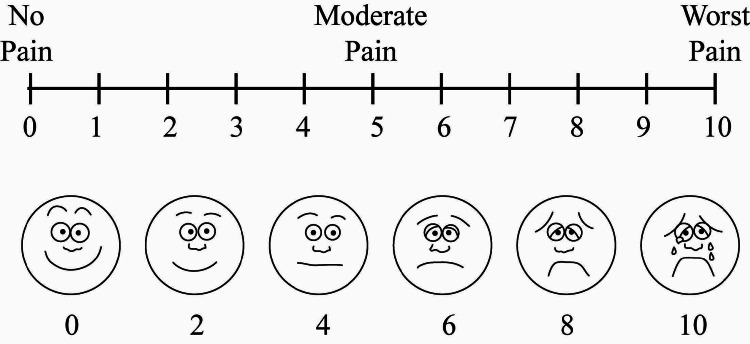
Visual Analogue Scale

The VAS is considered the most appropriate tool because of its simplicity; less than a minute is sufficient for recording and no special training is required hence many authors already used it in their studies [4,5]. It was used to assess the patient's level of pain and level of satisfaction in the present study. The insertion of the needle served as the stimulus for pain. The participants were asked to indicate their pain level on a 10-centimeter scale, and the operator recorded the nearest whole number. The pain scale ranged from 0 (no pain) to 10 (worst pain) which is depicted in Figure 3. The VAS satisfaction score, which included both extreme descriptors of satisfaction, was used to evaluate patient satisfaction. On a scale from 0 to 10, where 0 represented no satisfaction at all and 10 represented total satisfaction, the patients indicated their level of satisfaction by drawing a vertical line. The VAS was chosen due to its ease and widespread usage as a standardized pain scoring system. The patient's pain perception was assessed by observing their behavior using the SEM (sound, eye, and motor) scale which is shown in Table 1. This scale is used to evaluate the connection between pain and the corresponding reactions exhibited by the patient's eyes, body motions, and verbal discomfort expression. A single clinician performed the entire procedure.

**Table 1:** Sound, eye, motor (SEM) scale

Parameter	Comfort (1)	Mild Discomfort (2)	Moderate Discomfort (3)	Severe Discomfort (4)
Sound	No sound	Non-specific sound	Verbal complaint, louder sound	Verbal complaints, shouting, crying
Eye	No sign	Dilated eyes without tears (anxiety sign)	Tears, sudden eye movements	Crying tears covering the face
Motor	Relaxed body and hand status	Muscular contraction, contraction of hands	Sudden body and hand movement	Hand movement for defense, turning the head to the opposite side

Statistical analysis

Statistical analysis was done by Statistical Package for Social Sciences (SPSS) for Windows version 20.0. (IBM Corp., Armonk, NY) and Microsoft Excel v. 2007. Quantitative variables were summarized using the mean and standard deviation, while categorical variables were summarized using frequency and percentage. The primary outcomes, which include the mean scores of VAS pain, VAS satisfaction, and SEM, were compared between the two groups using the Mann-Whitney U test. A p-value < 0.05 was considered statistically significant. 

## Results

Twenty patients were enrolled in the study; hence 40 sites were equally allocated between Group 1 and Group 2 according to randomization. Among the participants, 14 (70%) were male, with ages ranging from 35 ± 5 years and six (30%) were female with ages ranging from 42 ± 5 years. The commonest extracted tooth in patients was the mandibular right third molar tooth (20%) and the mandibular left second molar tooth (7%) as shown in Table 2.

**Table 2:** Demographic details of the study group

	Number of participants	Age
Total number of participants	20	38.5 years
Male	14	35 ± 5 years
Female	6	42 ± 5 years

Visual analogue scale 

The study results revealed that Group 1 mean VAS pain score was 2.4 ± 0.44 and Group 2 mean VAS pain score was 3.0 ± 0.44 (mean ± standard deviation), respectively. This difference was found to be statistically significant (p = 0.001). The pain score demonstrates that Group 1 experienced minimal pain compared to Group 2. In addition, it was also found that the mean patient satisfaction score for Group 1 was 9.8 ± 0.22 and Group 2 was 9.2 ± 0.40. This difference in pain satisfaction between the two groups was statistically significant (p = 0.003). This suggests that Group 1 was more satisfied than Group 2. 

SEM scale

According to the statistical study, Groups 1 and 2 had mean SEM scale values of 1.1 ± 0.1 and 1.30 ± 0.46 (mean ± standard deviation), respectively. This difference was found to be statistically significant (p = 0.016). The analysis indicated that Group 1 had a higher level of comfort compared to Group 2, as reflected by a lower score for pain perception on the SEM scale for Group 1. This difference was statistically significant, highlighting the superior comfort experienced by Group 1 patients. Both the group's mean values of the primary outcome variables are shown in Table 3. 

**Table 3:** Primary outcome variables between the two groups VAS: visual analogue scale; SEM scale: sound, eye, motor scale; SD: standard deviation, * p < 0.05 considered as statistically significant

S. no	Primary Outcomes	Group	Mean value ± SD	p-value
1	Pain level using VAS score	Group 1	2.4± 0.44	0.001*
		Group 2	3.0± 0.44	
2	Patient satisfaction using VAS	Group 1	9.8± 0.22	0.003*
		Group 2	9.2± 0.40	
3	Pain perception using SEM scale	Group 1	1.1± 0.1	0.016*
		Group 2	1.30± 0.46	

## Discussion

When undergoing dental extractions, patients frequently struggle with issues such as dental anxiety and a fear of needles. A medical condition known as needle phobia, which affects approximately 10% of the population, has been linked to a variety of changes in a person's physiology, including variations in blood pressure, heart rate, electrocardiogram, and stress hormones [6]. A number of different strategies have been suggested as potential ways to lessen the discomfort that patients feel during dental procedures that require the use of local anesthesia injections. One widely adopted strategy is desensitizing the injection site. Techniques such as transcutaneous electronic nerve stimulation (TENS), the application of a topical anesthetic, topical cooling, computerized injection systems, pressure administration, and the use of an EMLA have been suggested [7]. Since the 1980s, dermatologists have been making use of EMLA cream, which is a mixture of prilocaine (2.5%) and lidocaine (2.5%), as a topical anesthetic [7]. Studies have shown that EMLA is very effective at reducing the amount of pain that is experienced during the process of needle insertion [7,8].

Cryoanesthesia is an additional method for the elimination of pain. This technique involves blocking local neural transmission by cooling the area that is experiencing pain. The application of refrigerant sprays and the utilization of ice are both viable options for accomplishing this goal. Ice application for the purpose of pre-cooling has been used for achieving anesthesia in the local site and analgesic effects for thousands of years [9]. A study that was carried out by Hindocha and colleagues discovered that the pain-relieving effects of ice were comparable to those of lidocaine gel, which was used as topical anesthesia [10]. The research was conducted at the point where the needle was inserted, and ice was said to be a more practical and affordable alternative to lidocaine gel [10]. Previous studies have demonstrated that pre-cooling can effectively alleviate the pain that is associated with injections [3,11-13].

Our study results found that patients who had undergone EMLA had significantly less pain and reported a higher level of overall satisfaction than those who had undergone ice treatment. Between the two groups, the pain difference (p = 0.001) and the patient satisfaction score difference (p = 0.003) were statistically significant. These findings are in accordance with the studies carried out by Vickers and Punnia-Moorthy as well as Pere et al.; in both studies, it was found that EMLA cream containing 5% was the most effective topical anesthetic for reducing the amount of pain that was experienced during the insertion of a needle [14,15]. Studies have shown that the efficacy of EMLA cream is significantly higher than that of the efficacy of other topical anesthetics [16,17].

The patient's response to stimulation was evaluated in our study using the SEM scale, which measures the patient's reactions in terms of sound, eye, and motor reactions. This allowed us to get a more accurate picture of the patient's overall level of comfort. The comparison of the EMLA and ice groups' levels of comfort in terms of pain perception revealed a distinction that was statistically significant between the two categories (p = 0.016). The study reveals that EMLA demonstrates a significant advantage over ice in diminishing pain and enhancing patient contentment. Surprisingly, the clinical evaluation scores for both EMLA and ice groups exhibited minimal disparity concerning pain and satisfaction. Nonetheless, it is worth noting that ice remains a viable and cost-effective substitute for EMLA cream, readily accessible for use [18]. The unpalatable taste of EMLA, the cost factor, and the potential risk of an anaphylactic reaction are some of the drawbacks of EMLA. 

The patients and the operator both felt the cold during pre-cooling, so it was impossible to blind either of them to the results of the study. This was one of the limitations of our investigation. The use of cream also made it difficult to achieve the desired effect of blinding. Because the effects of the topical anesthesia produced by ice wore off more quickly than expected, the injection of the local anesthetic had to be done more quickly [19]. In our research, we found that pain perception can be affected by a variety of environmental factors and that there are also differences in pain perception between the sexes, with females typically having stronger pain perceptions than males. The future area of research on this topic can be done on a larger sample size to find out the relationship between the different gauze needles with topical anesthetics like EMLA or ice, and different nerve block techniques between EMLA and ice. 

## Conclusions

The study results revealed that EMLA has a significant advantage over ice in terms of lower levels of pain, more patient satisfaction, and higher comfort level. EMLA can be considered the first choice of topical anesthetics, however, ice is recommended in resource-constrained dental set-ups as it is cost-effective.
